# Induction therapy and outcome of proliferative lupus nephritis in the top end of Northern Australia – a single centre study retrospective study

**DOI:** 10.1186/s12882-022-02849-w

**Published:** 2022-07-04

**Authors:** Chi Xu, Kim Ling Goh, Asanga Abeyaratne, Kelum Priyadarshana

**Affiliations:** 1 Department of Nephrology, Division of Medicine, Royal Darwin Hospital, Darwin, NT Australia; 2 Department of Renal Medicine, Royal Darwin Hospital, Rockland Drive, Tiwi, NT 0810 Australia; 3 Flinders University and Northern Territory Medical Program, Royal Darwin Hospital Campus, Darwin, NT Australia

**Keywords:** Lupus nephritis, Systemic lupus erythematosus, Mycophenolate, Indigenous Australians, Induction immunosuppression

## Abstract

**Background:**

Lupus nephritis is a common manifestation of Systemic Lupus Erythematosus. Mycophenolate is recommended by guidelines for induction therapy in patients with proliferative lupus nephritis and nephrotic range proteinuria Class V lupus nephritis. Indigenous Australians suffer disproportionally from systemic lupus erythematosus compared to non-Indigenous Australians (Anstey et al., Aust N Z J Med 23:646–651, 1993; Segasothy et al., Lupus 10:439–444, 2001; Bossingham, Lupus 12:327–331, 2003; Grennan et al., Aust N Z J Med 25:182–183, 1995).

**Methods:**

We retrospectively identified patients with newly diagnosed biopsy-proven class III lupus nephritis, class IV lupus nephritis and class V lupus nephritis with nephrotic range proteinuria from 1^st^ Jan 2010 to 31^st^ Dec 2019 in our institution and examined for the patterns of prescribed induction therapy and clinical outcome. The primary efficacy outcome of interest was the incidence of complete response (CR) and partial response (PR) at one-year post diagnosis as defined by the Kidney Disease: Improving Global Outcome (KDIGO) guideline. Secondary efficacy outcome was a composite of renal adverse outcome in the follow-up period. Adverse effect outcome of interest was any hospitalisations secondary to infections in the follow-up period. Continuous variables were compared using Student’s t-test or Mann–Whitney U-test. Categorical variables were summarised using frequencies and percentages and assessed by Fisher’s exact test. Time-to-event data was compared using the Kaplan–Meier method and Log-rank test. Count data were assessed using the Poisson’s regression method and expressed as incident rate ratio.

**Results:**

Twenty of the 23 patients included in the analysis were managed with mycophenolate induction upfront. Indigenous Australian patients (*N* = 15), compared to non-Indigenous patients (*N* = 5) received lower cumulative dose of mycophenolate mofetil over the 24 weeks (375 g vs. 256 g, *p* < 0.05), had a non-significant lower incidence of complete remission at 12 months (60% vs. 40%, *p* = 0.617), higher incidence of composite renal adverse outcome (0/5 patients vs. 5/15 patients, *p* = 0.20) and higher incidence of infection related hospitalisations, (incident rate ratio 3.66, 95% confidence interval 0.89–15.09, *p* = 0.073).

**Conclusion:**

Mycophenolate as upfront induction in Indigenous Australian patients were associated with lower incidence of remission and higher incidence of adverse outcomes. These observations bring the safety and efficacy profile of mycophenolate in Indigenous Australians into question.

## Introduction

Systemic lupus erythematosus (SLE) is a chronic multi system autoimmune disease that predominantly affects women of childbearing age. The disease often involves the kidneys with lupus nephritis (LN) occurring in approximately 50% of patients with SLE [1, 2]. Histologically, LN is classified into six classes depending on the glomerular pathology with proliferative LN divided to class III and class IV depending on whether the lesion is focal or diffused. Membranous LN is classified as class V LN [3]. Proliferative lupus nephritis and membranous LN with nephrotic range proteinuria are typically managed with a short and intensive induction phase treatment with systemic immunosuppression for 3 to 6 months [4–8].

In addition to corticosteroids, large multicenter randomised control trials (RCT) had supported the routine use of intravenous and oral cyclophosphamide as well as mycophenolate mofetil (MMF) for induction [1, 9–11]. These had emerged as the standard of care for upfront induction therapy. Rituximab, the anti-CD20 monoclonal antibody, was typically reserved for relapsed or refractory cases. In the phase 3 Lupus Nephritis Assessment with Rituximab (LUNAR) trial, the addition of rituximab to standard of care failed to demonstrate any additional benefit when compared to standard of care alone, despite a statistically significant improvement in complement C3 and C4 levels and a reduction in anti-double stranded DNA (anti-dsDNA) titer.

Aboriginal and Torres Strait Islander Australians (hereto respectively referred to as Indigenous Australians) are the first nation people of Australia. They suffered from a disproportionally higher incidence and prevalence of SLE and LN compared to non-Indigenous Australians [12–15]. Emerging evidence had suggested that SLE in Indigenous Australians could be pathologically distinct and manifest differently compared to SLE in non-Indigenous Australians [13, 15–17]. Currently, there are a paucity of data concerning the management of LN in Indigenous Australian patients. Despite observable differences in phenotype and biochemical markers between Indigenous Australians and non-Indigenous Australians LN, induction treatment had largely been extrapolated from studies of international cohorts and comparative to that of non-Indigenous Australians. We therefore sought to investigate the pattern of prescription and outcome in LN patient cohort from the Top End of Northern Territory, Australia and see if there is a difference in outcome between Indigenous Australian patients and non-Indigenous patients.

The Top End is a geographical region located in the northern tip of Northern Territory. It encompasses an area approximately 245,000 km2 that includes the capital city Darwin, Kakadu National Park, Arnhem Land, and Katherine Region. The Top End Health Service has a catchment area that occupies 35% of total land area of NT and 81% of total NT population. 26% of residents in the TEHS catchment area are Indigenous Australians [18].

## Methods

We retrospectively identified all patients with newly diagnosed and biopsy proven class III LN, class IV LN and class V LN with nephrotic range proteinuria from 1^st^ January 2010 to 31^st^ December 2019 in our institution. We then examined the pattern of prescribed immunosuppression therapy and the subsequent outcome during the follow-up period of 1^st^ January 2010 to 31^st^ December 2019.

Patients were identified from existing renal biopsy database. The database contained all renal biopsies performed in our institution from 2007. Two independent renal anatomical pathologists from an interstate laboratory examined and reported all biopsy samples. Histological data were collected thereafter from authorized reports. A diagnosis of LN and International Society of Nephrology/Renal Pathology Society (ISN/RPS) classification were made on both histological features as well as the presence of classic ‘full-house’ pattern of immunofluorescence staining to IgG, IgM, IgA, C3, C1q, Kappa and Lambda light chain. We included all patients > 18 years of age with newly diagnosed class III, IV and/or nephrotic-range proteinuric class V for analysis. Nephrotic range proteinuria is defined by presence of > 3 g urinary protein over 24 h collection. In cases where a 24 h urine collection was unavailable, nephrotic range proteinuria was defined by a spot albumin-creatinine ratio (ACR) > 300 mg/mmol. Patients were excluded if a history of LN was known prior to 1st January 2010.

The exposures of interest were the induction immunosuppression therapy received in the first 24 weeks since biopsy. Data were obtained from pharmacy record in our institution. Methylprednisolone, typically given in intravenous pulse therapy of 1 g repeated over 3 to 5 days, was recorded as a dichotomous variable. Cumulative prescribed dosages of oral prednisolone and MMF over the six months period were recorded and expressed as continuous variables. The MMF dose equivalent was recorded in patients that were prescribed mycophenolic acid (MPA), for example 360 mg MPA were recorded as 500 mg MMF and 720 mg MPA was recorded as 1000 mg MMF.

The primary efficacy outcome of interest was the incidence of CR and PR at one-year post diagnosis as defined by the Kidney Disease: Improving Global Outcome (KDIGO) guideline (See Table 1). Secondary efficacy outcome was a composite of renal adverse outcome consisting of relapse of disease as deemed by the treating clinician, doubling of serum creatinine for 3 months, end-stage kidney disease (ESKD) requiring dialysis for at least 12 sessions or death from any cause. The differences in primary and secondary outcomes were compared between Indigenous Australian and non-Indigenous Australian lupus nephritis patients. This was recorded as time-to-event data. Time at risk was measured using patient-years (ptyr), with 1 patient-year defined as an at-risk period of 1 year for 1 patient. Adverse effect outcome of interest was any hospitalisations secondary to infections, recorded as count data. The infection diagnosis was defined and recorded according to the primary admission diagnosis on discharge summary. Admissions were not included in the analysis if an infectious cause was the secondary diagnosis. Patient ethnicity, including whether they self-identified as Aboriginal and Torres Strait Australians or non-Indigenous Australians was obtained from the registration questionnaire at the time of first presentation to our institution and recorded as a dichotomous variable.

**Table 1:** KDIGO Clinical Response Criteria

Complete response (CR)	Decline in urine PCR to ≤ 0.5 g/g (≤ 50 mg/mmol); return of serum Cr previous baseline
Partial response (PR)	> 50% decrease in urine PCR; if there was nephrotic-range proteinuria, then reduction to < 3,000 mg/g [< 300 mg/mmol] AND; stabilization (± 25%), or improvement of serum Cr, but not to normal
No response	Failure to achieve CR or PR

*PCR* protein-creatinine ratio, *Cr* creatinine

Continuous variables were assessed for normality. Continuous variables were summarised using mean and standard deviation and compared using Student’s t-test if normally distributed; If not normally distributed, they were summarised using medians and interquartile ranges and compared using Mann–Whitney U-test. Categorical variables, including binary and ordinal outcomes, were summarised using frequencies and percentages and assessed by Fisher’s exact test. Time-to-event data was assessed using the Kaplan–Meier method. Count data, including the number of hospitalisations was assessed using the Poisson’s regression method and expressed as incident rate ratio. Statistical significance was defined by a two-tail *p*-value of < 0.05. All statistical analyses were performed with Stata/IC16.0 software.

Ethics approval was obtained from the Human Research Ethics Committees of the Menzies School of Research and the Top End Health Service Research Governance Office, Northern Territory, Australia (Reference number HREC-2020–3869).

## Results

During the follow-up period, Thirty-one patients were diagnosed with class III/IV and nephrotic range class V LN. Eight patients were excluded as they had a prior diagnosis of lupus nephritis and treated previously. 23 newly diagnosed LN patients were included for the analysis.

Mycophenolate was the immunosuppression agent of choice in 20 of the 23 patients. Of the three remaining patients, all of whom were Indigenous Australians, one patient received four doses of intravenous 1 g rituximab (on day 1 and 8 followed by two additional doses six months later) by her treating rheumatologist and general medicine physician and remained in CR after 39 months until the end of follow-up period. One patient was diagnosed with LN in her first trimester of pregnancy and was treated with combination tacrolimus and azathioprine for 3 weeks before her care was transferred interstate. 1 patient was managed with prednisolone initially before becoming disengaged from our medical service and ultimately succumbed to E. coli bacteraemia two months later. No patients in our study received cyclophosphamide upfront. The baseline characteristics of the 20 patients who received mycophenolate induction therapy are listed in Table 2. At the time of diagnosis, a higher proportion of Indigenous Australian patients had co-morbidities including diabetes mellitus, hypertension and acute rheumatic fever/rheumatic heart disease, these were however, not statistically significant. The baseline serum creatinine was comparable between the two cohorts. While a higher pre-induction urine albumin-creatinine ratio was observed in Indigenous Australian patients, this was not statistically significant.

**Table 2:** Baseline characteristics of patients who received mycophenolate

	Non-Indigenous Australians (*N* = 5)	Indigenous Australians (*N* = 15)	*P*-value
Age (mean ± SD)	36.4 ± 6.50	36.2 ± 12.37	0.97
Women, n (%)	3 (60%)	13 (87%)	0.20
Diabetes mellitus, n (%)	0	3 (20%)	0.28
Hypertension n (%)	2 (20%)	3 (20%)	0.37
Acute rheumatic fever / rheumatic heart disease	0	4 (27%)	0.20
Pre-induction creatinine (μmol/L)	145.6 (± 131.4)	142.1 (± 92.25)	0.947
Pre-induction ACR (g/mol)	135.6 ± 187.7	341.1 ± 338.32	0.22
Pathological Classification			
III	1 (20%)	6 (40%)	0.25
IV	3 (60%)	5 (33%)	
V	0	2 (13%)	
III + V	0	2 (13%)	
IV + V	1 (20%)	0	

Table 3 lists the cumulative dose of immunosuppressive therapy received by 20 of the 23 patients that were managed with MMF/MPA upfront. 15 of the 20 patients that received MMF/MPA self-identified as Indigenous Australians. Compared to non-Indigenous Australians, Indigenous Australians received a significantly lower cumulative dose of MMF over the initial 24 weeks (374.6 g vs. 255.9 g, *p* = 0.047). The cumulative dosage of prednisolone over the initial 24 weeks and proportion of patients that received pulse methylprednisolone were similar.

**Table 3:** Induction therapy in patients received mycophenolate

	Total	Non-Indigenous Australians	Indigenous Australians	*P*-value
No. of Patients	20	5	15	
Mean eGFR^a^ (ml/min/1.73 m^2^)		76 (± 41.4)	75.3 (± 43.1)	0.976
Mean Creatinine (μmol/L)	143.0 (± 22.26)	145.6 (± 131.4)	142.1 (± 92.25)	0.947
Mean ACR (g/mol)	289.71 (± 70.7)	135.6 (± 187.7)	341 (± 338.3)	0.217
Total MMF dose (g, mean ± SD)	285 ± 117.57	374.6 ± 97.4	255.9 ± 110.79	0.047
Total Prednisolone dose (mg, mean ± SD)	3735 ± 1959	3734 ± 1674.2	3736 ± 2100.3	0.999
Number of patients received Pulse methylprednisolone	8 (40%)	3 (60%)	5 (33%)	0.347

MMF – Mycophenolate mofetil; SD – standard deviation; ACR – albumin-creatinine ratio

^a^eGFR obtained utlising the CKD-EPI equation [19]

Table 4 illustrates the rate of remission at one-year post commencement of mycophenolate induction therapy. While a lower rate of CR was achieved among patients self identify as Indigenous Australians, this was not statistically significant (40% vs. 60%, *p* = 0.617). The combined remission rate was similar between the two groups.

**Table 4:** Remission rate as per KDIGO criteria

	Total	Non-Indigenous Australians	Indigenous Australians	*P*-value
No. of Patients	20	5	15	
CR	9 (45%)	3 (60%)	6 (40%)	0.617
CR + PR	15 (75%)	4 (80%)	11(73%)	1

**Fig. 1:**
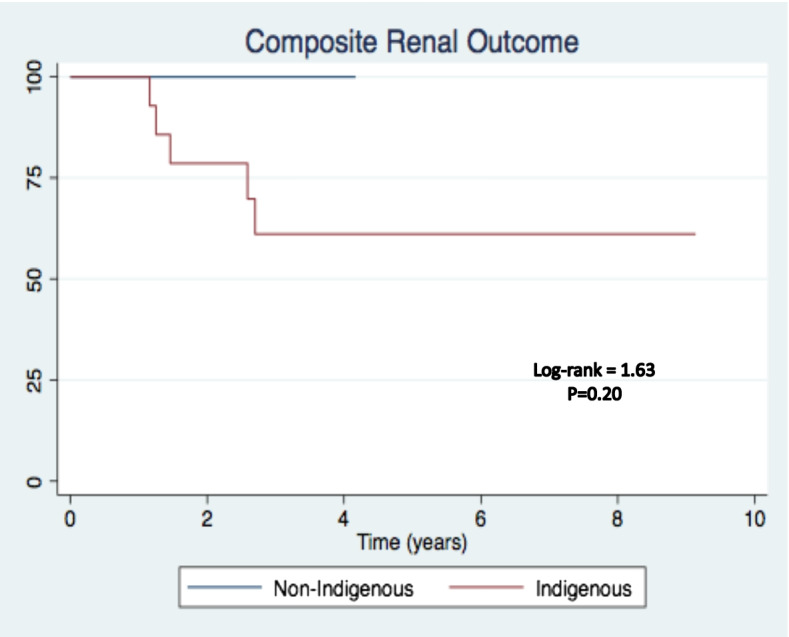
Kaplan–Meier analysis of composite adverse renal outcome in patients received Mycophenolate

The composite renal adverse outcome of disease relapse, doubling of creatinine, dialysis dependence and death occurred in 5 of the 15 Indigenous Australian patients. The composite renal adverse outcome did not occur in any of the non-Indigenous patients.. Table 5 and Fig. 1 demonstrate the composite outcome and the Kaplan–Meier analysis.

**Table 5:** Composite renal adverse outcome in patients received mycophenolate

	Non-Indigenous Australians	Indigenous Australians
Total number	5	15
Death	0	2
Relapse	0	4
Doubling of creatinine	0	5
Dialysis dependence	0	4

**Fig. 2:**
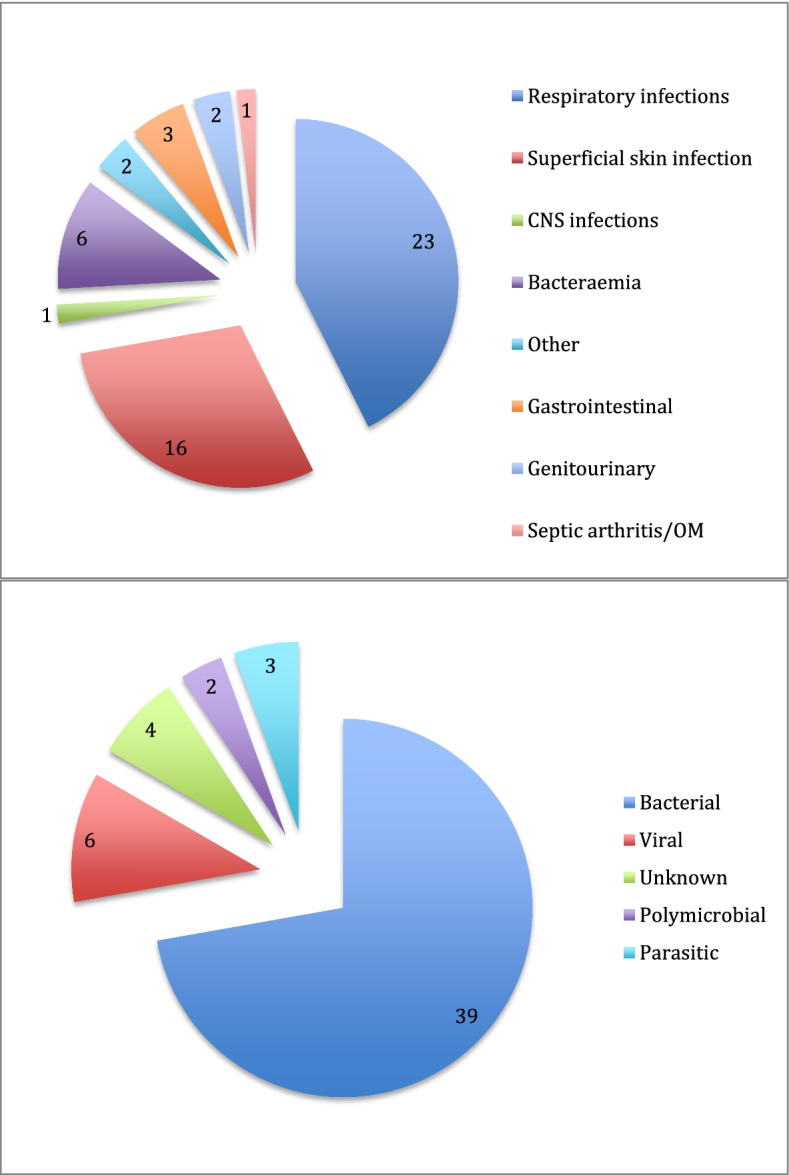
Aetiology of infections organ system (Top) and organisms (Bottom)

Table 6 summarised the incidence and incidence rate ratio of infection related hospitalisations and infection related intensive care unit (ICU) admissions over the follow-up period. Overall, 46 episodes of infection related hospitalisations occurred in patients who received mycophenolate. 96% of the hospitalisation episodes occurred in Indigenous Australians. This equated to a non-statistically significant incidence rate ratio of 3.66 (95%CI 0.89—15.09, *p* = 0.073). The incidence rate of ICU admissions was also not statistically significant, IRR = 1.16 (95%CI 0.14 – 9.45, *p* = 0.599). Figure 2 illustrates the aetiology of infection observed. Respiratory infectious were the most common infectious seen in Indigenous Australian patients treated with mycophenolate, occurring in 23 of the 44 episodes. This was followed by superficial skin infection, occurring in 16 episodes, and bacteraemia, occurring in 6 episodes. 8 episodes of hospitalisations had more than one site of infection. Notable infections included Nocardia intracranial abscess in one, grade 2/3 crusted scabies in three, and melioid bacteraemia in two. In one case of melioid bacteraemia was complicated with septic shock and multiple hepatic abscesses.

**Table 6:** Incidence of infection related hospitalisations over the follow-up period in patients treated with mycophenolate

	Total	Non-Indigenous	Indigenous Australians	IRR^b^ (95% CI)	*P*-value
Total number	20	5	15		
Time at risk (ptyr^a^)	87.55	12.48	75.07		
Infection related admissions	46	2^c^	44		
Incidence rate (per ptyr)	0.53	0.16	0.59	3.66 (0.89—15.09)	0.073
Infection related ICU admissions	8	1	7		
Incidence rate (per ptyr)	0.09	0.08	0.09	1.16 (0.14 – 9.45)	0.599

^a^*Ptyr* Patient-year, ^b^*IRR* Incidence rate ratio, ^c^One patient had VZV PCR positive shingles, another patient had infective colitis while in Singapore and was admitted to ICU with septic shock

## Discussion

This retrospective comparative study adds to our existing knowledge of the induction treatment and outcome of lupus nephritis in Australia. It is, to our knowledge, the first study examining and comparing the differences in outcome between Indigenous Australian and non-Indigenous Australian lupus nephritis patients of Northern Territory.

MMF was the most frequently prescribed initial induction immunosuppressive agents in 20 of the 23 patients. The routine use of mycophenolate as first-line agent for induction therapy is in keeping with recommendations from society guidelines. The American College of Rheumatology, European League Against Rheumatism and Kidney Disease: Improve Global Outcome guidelines recommended the use of MMF/MPA or low dose cyclophosphamide as reasonable first line therapy for induction [4–7], with a recommended target dose of 2-3 g daily (336-504 g cumulative over 24 weeks. Preferential use of MMF/MPA over low-dose cyclophosphamide in our unit was an established practice based on anecdotal evidence suggesting of higher risk of neutropenia and infectious complications associated with cyclophosphamide.

When compared to non-Indigenous Australians, Indigenous Australians were less likely to be in CR at 12 months and had a higher incidence of composite adverse renal outcomes over the follow-up period. The poor rate of CR observed in Indigenous Australians were consistent with those reported in the literature [20, 21]. The reason for the observed poor rate of CR and higher incidence of composite adverse renal outcomes were likely multifactorial. In addition to receiving a lower cumulative dose of MMF over induction period, lower socio-economic status, higher prevalence of co-morbid conditions such as diabetes and geographical barrier to healthcare were likely to have all contributed to the observed worse outcome. When compared to non-Indigenous Australians, Indigenous Australians of the Northern Territory were more likely to be residing in remote communities, occupy the lowest quintile of equivalised weekly household income and were less likely to have access to health provider when necessary [22]. These factors may have also contributed to late presentation of disease, leading to delay in diagnosis and commencement of treatment.

A higher incidence of hospitalisation secondary to infections was observed in Indigenous Australian patients in our study. This is in spite of the routine use of trimethoprim-sulfamethoxazole antimicrobial prophylaxis for melioidosis and Pneumocystis pneumonia in our centre [23]. The higher incidence of infection in immunosuppressed Indigenous Australians was consistent with observations from other comparative studies in Indigenous LN patients and in renal transplant recipients [21, 24]. As an example, Ghazanfari et al. reported infections as the leading cause of mortality in their cohort of Indigenous Australian LN patients, occurring in 38% of death [25]. The observed incidence of infection appeared to exceed those of comparative cohorts in other studies. One randomised control trial comparing MMF to intravenous cyclophosphamide reported 2 episodes of infection requiring hospitalization in their cohort of 33 patients in the intervention arm who received MMF, and a corresponding incidence of 1 per 234 patient-months [26]. Another randomized control trial reported 41 episodes of infection over 1738 patient-weeks of follow up but only one episode of severe infection in their cohort of 71 patients randomised to MMF [27]. The higher incidence of infection observed in our study may have led to the lower cumulative dose of mycophenolate prescribed to Indigenous Australian patients, whether deliberate or reactionary. Elevated existing background risk of infection including pneumococcal disease [28], group A Streptococcus infection [29], strongyloidiasis [30], and tuberculosis [31] in Indigenous Australians are known and documented in literature [32]. The incidence of Acute Rheumatic Fever in Indigenous Australians is near a hundred times that of non-Indigenous Australians [33]. Despite significant improvement in the incidence of culture-confirmed tuberculosis from 1989 to 2019, this far exceeds the incidence of Australian-born non-Indigenous Australians [34]. In one prospective cohort study conducted in our institution, an annualised incidence of 40.8 admissions per 1000 population for general admission and 4.7 per 1000 ICU admission was reported in Indigenous Australians, a near four-fold increase in sepsis-related admission and ICU admission compared to non-Indigenous Australians [35]. The incidence of infection related hospitalisation of 0.59 per patient-year reported by our study also exceeded these figures in non-immunosuppressed Indigenous Australians; further highlighting the complexity associated with the management of chronic autoimmune disorders in this infection-susceptible population. A careful consideration of the competing risk of autoimmune disease activity and background infection risk is necessary when considering the optimal agent used for induction therapy.

Targeted immunosuppressive therapy aimed at suppressing specific pathogenic humoral immunity pathways could an alternative approach to optimize LN outcome. The sparing of the innate immune system and cell-mediated adaptive immunity may potentially limit the risk of infection. The efficacy and safety of intravenous anti-CD20 + B cell depleting antibody rituximab for the induction treatment was previously examined in the LUNAR trial. Despite some promising biochemical results with statistically significant reduction in anti-dsDNA and improvement in complement C3 and C4 levels, the trial failed to achieve the primary end point [36]. Rituximab, since then, had largely been reserved for patients with refractory or relapsed disease [1, 4–6]. The safety and efficacy of rituximab in the Top End of Northern Territory was examined in a retrospective descriptive study examining the off-label use of rituximab in patients with autoimmune diseases [37]. The authors of the study reported 18 episodes of infection in 66 patients, of whom, 41(62.1%) self-identified as Indigenous Australians [37]. A ‘clinically significant response’ was seen in 3 of the 4 patients treated for Lupus Nephritis. Accepting the limitations of retrospective study and the inconsistent documentation of objective disease activity, the authors concluded that off-label use of rituximab for treatment of autoimmune diseases is common and safe in majority of cases. Further studies of rituximab for management of LN in the Top End are warranted to support its use as upfront therapy in Indigenous Australians.

Social inequities such as inadequate housing, overcrowding and poverty are also hypothesised to be key drivers of infection risk in Indigenous Australians [38–41]. In addition to optimising the induction therapy to balance the competing risk of autoimmune disease and adverse risks of infections, strategies aimed at improving LN outcome in Indigenous Australians of Northern Territory may also necessitate a system-wide approach aimed at improving disparities in social inequities and barrier to accessing healthcare in order to reduce exposure to background infection risks and diagnostic latency.

The major strength of our study is the unique population examined. To our knowledge, this is the first study examining the treatment and outcome of LN in an Indigenous Australian predominant patient cohort. Despite having a known higher incidence and prevalence of SLE and LN, Indigenous Australians are underrepresented in the existing medical literature. The major limitation of our study comes from its retrospective observational design. As data collection was highly reliant on hospital medical records, the lack of information and missing data, particularly pertaining to vaccination records makes the adjustment of potential confounders challenging. Missing data may have also contributed to the relatively small sample size of 23 patients in our study which would have significantly underpowered our study. A prospective study involving multiple geographical centres with a high proportion of Indigenous Australian patient population should therefore be considered in order to better understand the differences in treatment response between Indigenous Australian and non-Indigenous Australian LN patients.

## Conclusion

We retrospectively reviewed and compared the induction treatment and outcome of all patients with newly diagnosed proliferative LN and/or class V LN with nephrotic range proteinuria. In our cohort of 23 patients, mycophenolate was the most prescribed upfront induction therapy in 20 patients. Indigenous Australian LN patients, when compared to non-Indigenous LN patients, were prescribed a lower cumulative dosage of mycophenolate, had a non-significant lower incidence of CR at 1 year, and a higher incidence of composite renal adverse outcome including relapse, doubling of creatinine, need for long-term renal replacement and death. Indigenous Australian LN patients were also found to have a non-statistically higher incidence of infection related admissions despite a lower prescribed mycophenolate dose. These results bring in to question the safety and efficacy profile of mycophenolate as induction therapy in Indigenous Australian LN patients. Future studies should examine alternative approaches that minimize infection risk and improve renal outcomes in Indigenous Australian patients.

## Data Availability

The datasets used and/or analysed during the current study are available from the corresponding author on reasonable request.

## Abbreviations

SLE: Systemic lupus erythematosus
LN: Lupus nephritis
RCT: Randomised control trial
LUNAR: Lupus Nephritis Assessment with Rituximab study trial
CR: Complete remission
PR: Partial remission
MMF: Mycophenolate mofetil
Anti-dsDNA: Anti-double stranded DNA
ISN/RPS: International Society of Nephrology/Renal Pathology Society
ACR: Albumin-creatinine ratio
PCR: Protein-creatinine ratio
MPA: Mycophenolic acid
KDIGO: Kidney disease: improving global outcome
ESKD: End stage kidney disease
Ptyr: Patient-year
ICU: Intensive care unit

## References

[1] Parikh SV, Almaani S, Brodsky S, Rovin BH (2020). Update on Lupus Nephritis: Core Curriculum 2020. Am J Kidney Dis.

[2] Danchenko N, Satia JA, Anthony MS (2006). Epidemiology of systemic lupus erythematosus: A comparison of worldwide disease burden. Lupus.

[3] Bajema IM, Wilhelmus S, Alpers CE (2018). Revision of the International Society of Nephrology/Renal Pathology Society classification for lupus nephritis: clarification of definitions, and modified National Institutes of Health activity and chronicity indices. Kidney Int.

[4] Hahn BH, McMahon MA, Wilkinson A (2012). American College of Rheumatology guidelines for screening, treatment, and management of lupus nephritis. Arthritis Care Res..

[5] Fanouriakis A, Kostopoulou M, Cheema K (2020). 2019 Update of the Joint European League Against Rheumatism and European Renal Association-European Dialysis and Transplant Association (EULAR/ERA–EDTA) recommendations for the management of lupus nephritis. Ann Rheum Dis..

[6] Cattran DC, Feehally J, Cook HT (2012). Kidney disease: Improving global outcomes (KDIGO) glomerulonephritis work group. KDIGO clinical practice guideline for glomerulonephritis. Kidney Int Suppl..

[7] Disease R, Rheum A, Rheumatology C. KDIGO GN Guideline update – Evidence summary Lupus nephritis Antimalarial therapy for lupus nephritis. Published online 2018:1–7.

[8] Mok CC, Yap DYH, Navarra SV (2013). Overview of lupus nephritis management guidelines and perspective from Asia. Int J Rheum Dis.

[9] Austin HA, Klippel JH, Balow JE, et al. Therapy of lupus nephritis. Controlled trial of prednisone and cytotoxic drugs. N Engl J Med. Published online 1986. 10.1056/NEJM198603063141004 10.1056/NEJM198603063141004 3511372

[10] Appel GB, Contreras G, Dooley MA, et al. Mycophenolate mofetil versus cyclophosphamide for induction treatment of lupus nephritis. J Am Soc Nephrol. Published online 2009. 10.1681/ASN.2008101028 10.1681/ASN.2008101028 PMC2678035 19369404

[11] Houssiau FA, Vasconcelos C, D’Cruz D (2002). Immunosuppressive therapy in lupus nephritis: The Euro-Lupus Nephritis Trial, a randomized trial of low-dose versus high-dose intravenous cyclophosphamide. Arthritis Rheum..

[12] Anstey NM, Bastian I, Dunckley H, Currie BJ (1993). Systemic lupus erythematosus in Australian Aborigines: high prevalence, morbidity and mortality. Aust N Z J Med.

[13] Segasothy M, Phillips PA (2001). Systemic lupus erythematosus in Aborigines and Caucasians in central Australia: A comparative study. Lupus.

[14] Bossingham D (2003). Systemic lupus erythematosus in the far north of Queensland. Lupus.

[15] Grennan DM, Bossingham D (1995). Systemic lupus erythematosus (SLE): different prevalences in different populations of Australian Aboriginals. Aust N Z J Med.

[16] Vincent FB, Bourke P, Morand EF, Mackay F, Bossingham D (2013). Focus on systemic lupus erythematosus in Indigenous Australians: Towards a better understanding of autoimmune diseases. Intern Med J.

[17] Mason JA, Bossingham D (2009). The clinical characterisation of systemic lupus erythematosus in a Far North Queensland Indigenous kindred. Lupus.

[18] Department of Health NG. 2019–2020 Department of Health Annual Report. Published online 2020.

[19] Levey AS, Stevens LA, Schmid CH (2009). A new equation to estimate glomerular filtration rate. Ann Intern Med.

[20] Nigam A, Baer R, Green S, Neuen BL, Vile A, Mantha M (2020). Lupus nephritis in Indigenous Australians: a single-centre study. Intern Med J.

[21] Ghazanfari F, Jabbar Z, Nossent J (2018). Renal histology in Indigenous Australians with lupus nephritis. Int J Rheum Dis.

[22] Australian Institute of Health and Welfare. Aboriginal-and-Torres-Strait-Islander-Health-Performance-Framework-2017-report-Northern-Territory. Published online 2017.

[23] Majoni SW, Hughes JT, Heron B, Currie BJ (2017). Trimethoprim+Sulfamethoxazole Reduces Rates of Melioidosis in High-Risk Hemodialysis Patients. Kidney Int Rep.

[24] Boan P, Swaminathan R, Irish A (2017). Infectious complications in indigenous renal transplant recipients in Western Australia. Int Med J Published online.

[25] Ghazanfari F, Jabbar Z, Nossent J (2018). Renal histology in Indigenous Australians with lupus nephritis. Int J Rheum Dis.

[26] Chan TM, Tse KC, Tang CSO, Mok MY, Li FK (2005). Long-term study of mycophenolate mofetil as continuous induction and maintenance treatment for diffuse proliferative lupus nephritis. J Am Soc Nephrol.

[27] Ginzler EM, Anne Dooley M, Aranow C, et al. Mycophenolate Mofetil or Intravenous Cyclophosphamide for Lupus Nephritis. n engl j med. 2005;353. www.nejm.org. Accessed 30 Apr 2022. 10.1056/NEJMoa043731 16306519

[28] Meder KN, Jayasinghe S, Beard F (2020). Long-term impact of pneumococcal conjugate vaccines on invasive disease and pneumonia hospitalizations in indigenous and non-indigenous Australians. Clin Infect Dis.

[29] May PJ, Bowen AC, Carapetis JR (2016). The inequitable burden of group a streptococcal diseases in indigenous Australians: We need to fill evidence gaps and make clinical advances to reduce these diseases of disadvantage. Med J Aust.

[30] Johnston FH, Morris PS, Speare R (2005). Strongyloidiasis: A review of the evidence for Australian practitioners. Aust J Rural Health.

[31] Bright A, Denholm J, Coulter C, Waring J, Stapledon R (2018). Tuberculosis notifications in Australia, 2015–2018. Commun Dis Intell.

[32] Ioannides S, Beard F, Larter N (2018). Vaccine Preventable Diseases and Vaccination Coverage in Aboriginal and Torres Strait Islander People, Australia, 2011–2015. Commun Dis Intell.

[33] Acute-rheumatic-fever-and-rheumatic-heart-disease-in-Australia-2015–2019.

[34] Meumann EM, Horan K, Ralph AP, et al. Tuberculosis in Australia’s tropical north: a population-based genomic epidemiological study. The Lancet Regional Health – Western Pacific. 2021;15. 10.1016/j.lanwpc.2021.100229 10.1016/j.lanwpc.2021.100229 PMC8350059 34528010

[35] Davis JS, Cheng AC, McMillan M, Humphrey AB, Stephens DP, Anstey NM (2011). Sepsis in the tropical Top End of Australia’s Northern Territory: disease burden and impact on Indigenous Australians. Med J Aust.

[36] Rovin BH, Furie R, Latinis K (2012). Efficacy and safety of rituximab in patients with active proliferative lupus nephritis: The lupus nephritis assessment with rituximab study. Arthritis Rheum..

[37] Wongseelashote S, Tayal V, Bourke PF (2018). Off-label use of rituximab in autoimmune disease in the Top End of the Northern Territory, 2008–2016. Intern Med J.

[38] Tong SYC, McDonald MI, Holt DC, Currie BJ (2008). Global implications of the emergence of community-associated methicillin-resistant Staphylococcus aureus in Indigenous populations. Clinical Infect Dis.

[39] Bailie RS, Stevens MR, McDonald E (2005). Skin infection, housing and social circumstances in children living in remote Indigenous communities: testing conceptual and methodological approaches. BMC Public Health.

[40] Davidson L, Knight J, Bowen AC (2020). Skin infections in Australian Aboriginal children: a narrative review. Med J Aust.

[41] Colosia AD, Masaquel A, Hall CB, Barrett AM, Mahadevia PJ, Yogev R (2012). Residential crowding and severe respiratory syncytial virus disease among infants and young children: a systematic literature review. BMC Infect Dis.

